# Adoptive Transfer of siRNA *Cblb*-Silenced CD8^+^ T Lymphocytes Augments Tumor Vaccine Efficacy in a B16 Melanoma Model

**DOI:** 10.1371/journal.pone.0044295

**Published:** 2012-09-04

**Authors:** Reinhard Hinterleitner, Thomas Gruber, Christa Pfeifhofer-Obermair, Christina Lutz-Nicoladoni, Alexander Tzankov, Manfred Schuster, Josef M. Penninger, Hans Loibner, Günther Lametschwandtner, Dominik Wolf, Gottfried Baier

**Affiliations:** 1 Department of Pharmacology and Genetics, Medical University Innsbruck, Innsbruck, Austria; 2 Laboratory of Tumor Immunology, Tyrolean Cancer Research Institute, Innsbruck, Austria; 3 Institute of Pathology, University Hospital Basel, Basel, Switzerland; 4 Apeiron-Biologics AG, Vienna, Austria; 5 Institute of Molecular Biotechnology of the Austrian Academy of Sciences, Vienna, Austria; 6 Department of Hematology and Oncology, Medical University Bonn, Bonn, Germany; Saint Louis University School of Medicine, United States of America

## Abstract

The ubiquitin ligase Cbl-b is an established regulator of T cell immune response thresholds. We recently showed that adoptive cell transfer (ACT) of *cblb* ^−/−^ CD8^+^ T cells enhances dendritic cell (DC) immunization-mediated anti-tumor effects in immune-competent recipients. However, translation of *cblb* targeting to clinically applicable concepts requires that inhibition of *cblb* activity be transient and reversible. Here we provide experimental evidence that inhibition of *cblb* using chemically synthesized siRNA has such potential. Silencing *cblb* expression by *ex vivo* siRNA transfection of polyclonal CD8^+^ T cells prior to ACT increased T cell tumor infiltration, significantly delayed tumor outgrowth, and increased survival rates of tumor-bearing mice. As shown by *ex vivo* recall assays, *cblb* silencing resulted in significant augmentation of intratumoral T cell cytokine response. ACT of *cblb*-silenced polyclonal CD8^+^ T cells combined with DC-based tumor vaccines predominantly mediated anti-tumor immune responses, whereas no signs of autoimmunity could be detected. Importantly, *CBLB* silencing in human CD8^+^ T cells mirrored the effects observed for *cblb*-silenced and *cblb*-deficient murine T cells. Our data validate the concept of enhanced anti-tumor immunity by repetitive ACT of *ex vivo cblb* siRNA-silenced hyper-reactive CD8^+^ T cells as add-on adjuvant therapy to augment the efficacy of existing cancer immunotherapy regimens in clinical practice.

## Introduction

The potential to harness patientś immune system as cancer therapy is an emerging strategy. Accordingly, Sipuleucel-T (Provenge™), a dendritic cell (DC) vaccine loaded with an antigen/GM-CSF conjugate, is the first active immunization approach approved for treatment of hormone-refractory prostate cancer [1], [2]. On the other hand, adoptive cell therapy (ACT) with autologous T cells in order to enforce immune-mediated tumor cell killing has also shown promising results in the treatment of various types of cancer. As an example, ACT using *ex vivo* expanded T cells can induce tumor regression in patients with advanced melanoma [3], [4]. Alternatively, T cells transduced with tumor antigen-specific T cell receptor (TCR) transgenes have been used to treat patients with melanoma [5], [6] or B cell lymphoma [7], thereby bypassing the need to expand tumor-specific T cells *ex vivo*. Nevertheless, although tumor-specific immune responses are frequently observed, sustained clinical benefits are documented in only a small fraction of patients [8].

A major drawback of ACT applications is that they generally require laborious *ex vivo* expansion and/or genetic engineering procedures to generate a potent tumor-reactive CD8^+^ T cell phenotype. These interventions bear the risk of insertional mutagenesis, e.g. by inappropriate insertion of T cell receptor (TCR)-transgenic lentiviral vectors within proto-oncogenes [9], potentially causing T cell leukemogenesis. Moreover, the therapeutic efficacy of ACT appears to be limited by immune-evasion mechanisms within the tumor-bearing host, such as secretion of transforming growth factor beta (TGFβ) by the tumor microenvironment and/or accumulation of regulatory T cells (Treg), both of which severely dampen *in vivo* activation, expansion, and tumor homing of transferred tumor-reactive CD8^+^ T cells. It is therefore desirable to establish strategies that enhance effector functions of adoptively transferred CD8^+^ T cells *in vivo* but minimize the requirement for *ex vivo* manipulation of CD8^+^ T cells prior to adoptive transfer.

**Figure 1:**
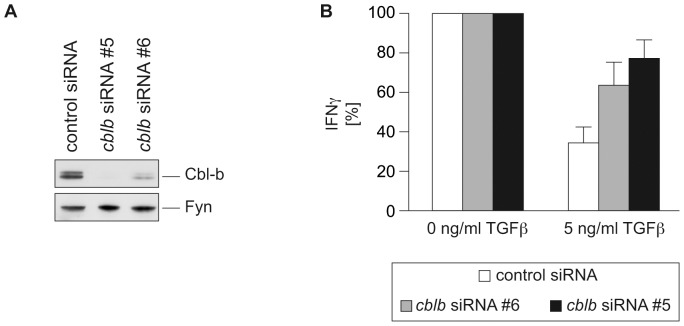
*Cblb* knockdown in CD8^+^ T cells confers resistance to the inhibitory effects of TGFβ. Mouse CD8^+^ T cells were nucleofected with *cblb* siRNA or control siRNA and stimulated with anti-CD3 and anti-CD28 in the presence or absence of TGFβ. The production of IFNγ by CD8^+^ T cells was efficiently suppressed by exposure to TGFβ while *cblb* knockdown cells still produced high levels of IFNγ. (A) Silencing efficacy of *cblb* siRNA was analyzed by immunoblotting. (B) Supernatants were taken two days post-transfection and the amount of IFNγ secreted was measured by BioPlex technology. IFNγ levels of each group without TGFβ were set 100%. Means ± SEM of three independent experiments are shown.

By using an *ex vivo* synthetic small interfering (si)RNA approach to inhibit “casitas B-lineage lymphoma proto-oncogene b (*cblb*)”, a member of the mammalian family of RING E3 ubiquitin ligases, we demonstrated this as a potentially rational approach to achieve such goals, as it profoundly improves the efficacy of ACT for cancer immunotherapy. Cbl family members have evolutionarily conserved roles in regulating protein tyrosine kinases [10]. Specifically, Cbl-b regulates adaptive immunity by setting activation thresholds of mature lymphocytes [11], [12], [13]. Loss of *cblb* expression renders animals susceptible to autoimmunity, and variants within the *CBLB* gene are associated with multiple sclerosis in humans [14]. Mechanistically, *cblb* deficiency uncouples CD3^+^ T cells from the requirement of CD28 co-stimulation for adequate activation via the TCR, establishing an active role of Cbl-b in the induction and maintenance of peripheral T cell tolerance [15], [16]. Moreover, *cblb*-deficient animals are less susceptible to tumor formation in induced as well as spontaneous mouse cancer models relevant for human cancers. In detail, *cblb*-deficient animals are able to reject implanted TC-1 lung fibroblast tumors [17], [18], EL-4 lymphomas [19], E.G7 lymphomas [20], B16ova melanomas [21], disseminated leukemia [22], mammary adenocarcinomas [23] as well as spontaneous UVB induced skin tumors [17], and genetically induced T cell lymphomas [20]. Studies show gene ablation of *cblb* in the CD8^+^ T cell compartment to be both necessary and sufficient for immunological rejection of malignant tumors in immune competent recipients [20], [21].

**Figure 2:**
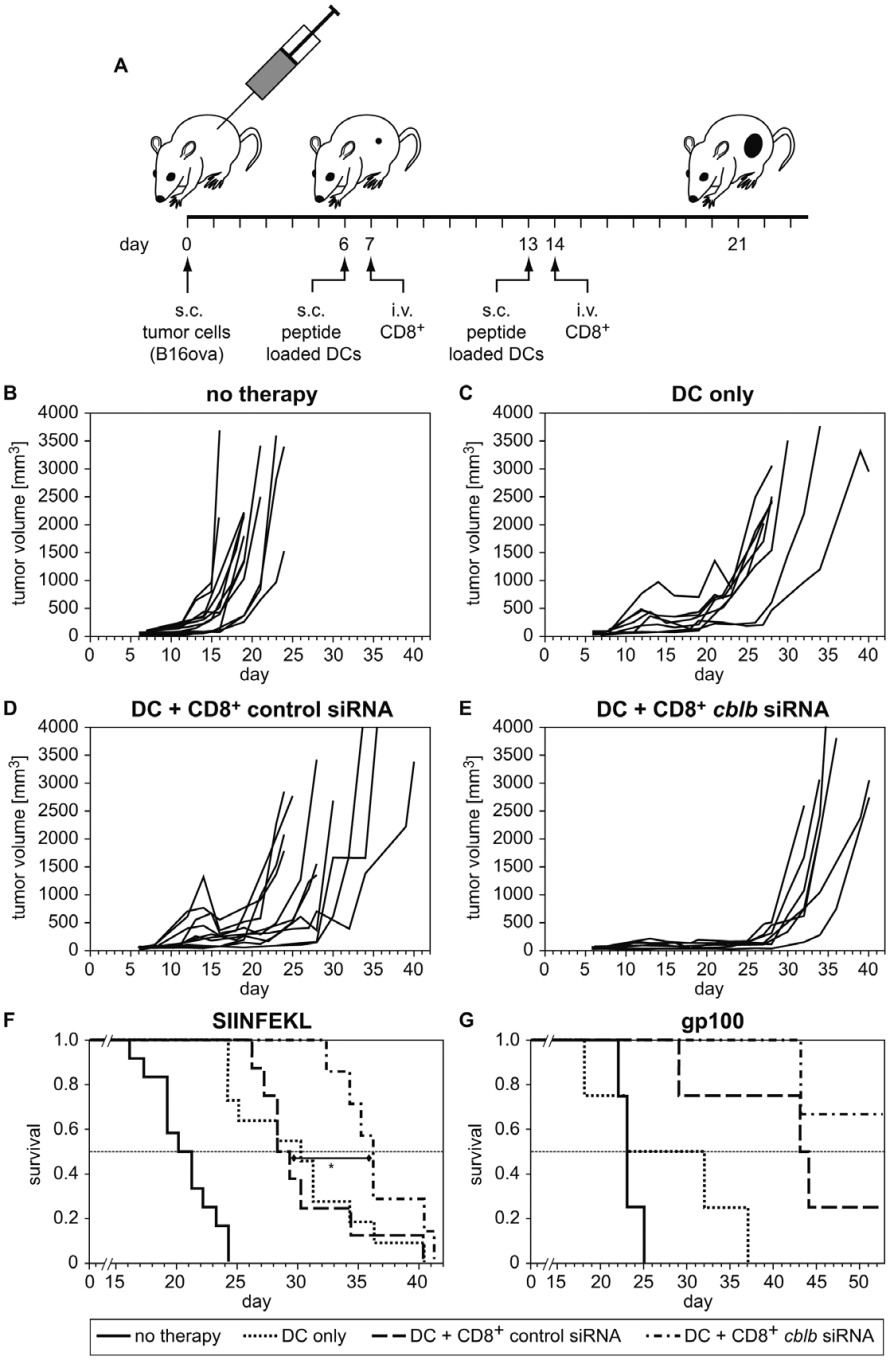
ACT of polyclonal *cblb*-silenced T cells reduces tumor growth rates *in vivo*. (A) Tumor therapy schedule. Wild type mice s.c. injected with 1×10^5^ B16ova cells were vaccinated with (B-F) SIINFEKL-pulsed DCs or (G) gp100-pulsed DCs on days 6 and 13. Twenty-four hours after DC vaccination, either control or *cblb* siRNA nucleofected polyclonal CD8^+^ T cells (5×10^6^) were i.v. injected into tumor bearing mice. (B) Tumor growth of untreated mice, (C) DC vaccinated mice, (D) mice treated with DCs plus control-siRNA nucleofected CD8^+^ T cells, and (E) DCs plus *cblb* siRNA silenced CD8^+^ T cells are shown. Tumor volume was measured every second day. Representative results out of two independent experiments are shown (n = 7 - 12 mice per group). (F) Survival of the animals described in (B–E) was monitored and the respective Kaplan-Maier curves are given. p = 0.03 (DC+CD8^+^ *cblb* siRNA versus DC+CD8^+^ control siRNA). (G) Survival of B16ova-challenged wild type mice treated as in (B-E) but vaccinated with gp100-pulsed DCs. Respective Kaplan-Maier curves are given (n = 3 - 4 mice per group).

Moreover, *cblb* deficiency renders CD8^+^ T cells hypo-responsive to the suppressive effects exerted by Treg via defects of the TGFβ receptor signaling pathway [19], [21], [24], [25].

Thus, genetic evidence from knockout animal studies suggests that inactivation of Cbl-b, as a non-redundant negative regulator of effector CD8^+^ T cell signaling, represents a rational approach to improve anti-cancer T cell reactivity *in vivo*. Consistently, ACT of *cblb-*deficient hyperactive polyclonal CD8^+^ T cells into fully immune-competent animals in combination with a dendritic cell (DC) vaccine markedly delayed tumor outgrowth and substantially improved survival rates [21]. As a proof of concept study for further clinical development of this strategy, we here establish synthetic *cblb* siRNA treatment of polyclonal CD8^+^ T cells prior to ACT as a therapeutic approach to elicit enhanced DC-based tumor vaccine efficacy. *Cblb*-silenced CD8^+^ T cells are hyper-responsive and mostly protected from the suppressor effects of TGFβ *in vitro*. This translated into a markedly increased anti-tumor efficacy without autoimmunity induction of *cblb*-silenced CD8^+^ T cells when combined with a DC vaccine. Thus our proof of concept study using *ex vivo* synthetic siRNA-mediated inactivation of *cblb* validates the concept of inhibiting Cbl-b (by siRNA prior to ACT or by systemic pharmacological antagonists) as a rational strategy to augment the effectiveness of adoptively transferred immune cells.

**Table 1:** Mean survival of B16ova melanoma bearing mice *(SIINFEKL-pulsed DCs).*

Therapy	Treatment Cycles	Mean Survival ± SEM (days)
no therapy (n = 12)	2	20±0.7
DC only (n = 11)	2	30±1.6
DC+CD8^+^ control siRNA (n = 8); a	2	30±1.6
DC+CD8^+^ *cblb* siRNA (n = 7); a,b	2	36±1.2
DC+CD8^+^ *cblb* siRNA (n = 4); b	3	41±0.5

a p = 0.03 (DC+CD8^+^ control siRNA versus DC+CD8^+^ *cblb* siRNA; 2 treatment cycles).

b p = 0.051 (DC+CD8^+^ *cblb* siRNA; 2 treatment cycles versus 3 treatment cycles).

**Table 2:** Mean tumor volume of B16ova melanoma bearing mice *(gp100-pulsed DCs).*

Therapy	day 14	day 21	day 28
no therapy	190±44	1199±103	
DC only	126±91	472±412	
DC+CD8^+^ control siRNA	22±14	137±115	663±640
DC+CD8^+^ *cblb* siRNA	11±7	16±15	54±50

Mean tumor volume [mm^3^] ± SEM.

## Results

### Transient *cblb-*silencing via Synthetic siRNA Reduces TGFβ Sensitivity *in vitro* and Induces Enhanced Anti-tumor Effects *in vivo*

We first established synthetic siRNA that efficiently targets *cblb* in murine CD8^+^ T cells. Two non-overlapping siRNA oligonucleotides reduced Cbl-b expression in primary mouse CD8^+^ T cells, albeit one (#6) to a lesser extent (Figure 1A). TGFβ is a major immunosuppressive cytokine in the tumor environment and Cbl-b was demonstrated to mediate at least some of its effects [19]. We therefore tested the *in vitro* sensitivity of *cblb*-silenced CD8^+^ T cells towards TGFβ. As a result, these cells are partially resistant to TGFβ treatment, which is in accordance with the results obtained with T cells genetically deficient in *cblb* [20], [21] (Figure 1B). Of note, the extent of this partial resistance corresponds with the efficacy of the respective siRNA.

**Figure 3:**
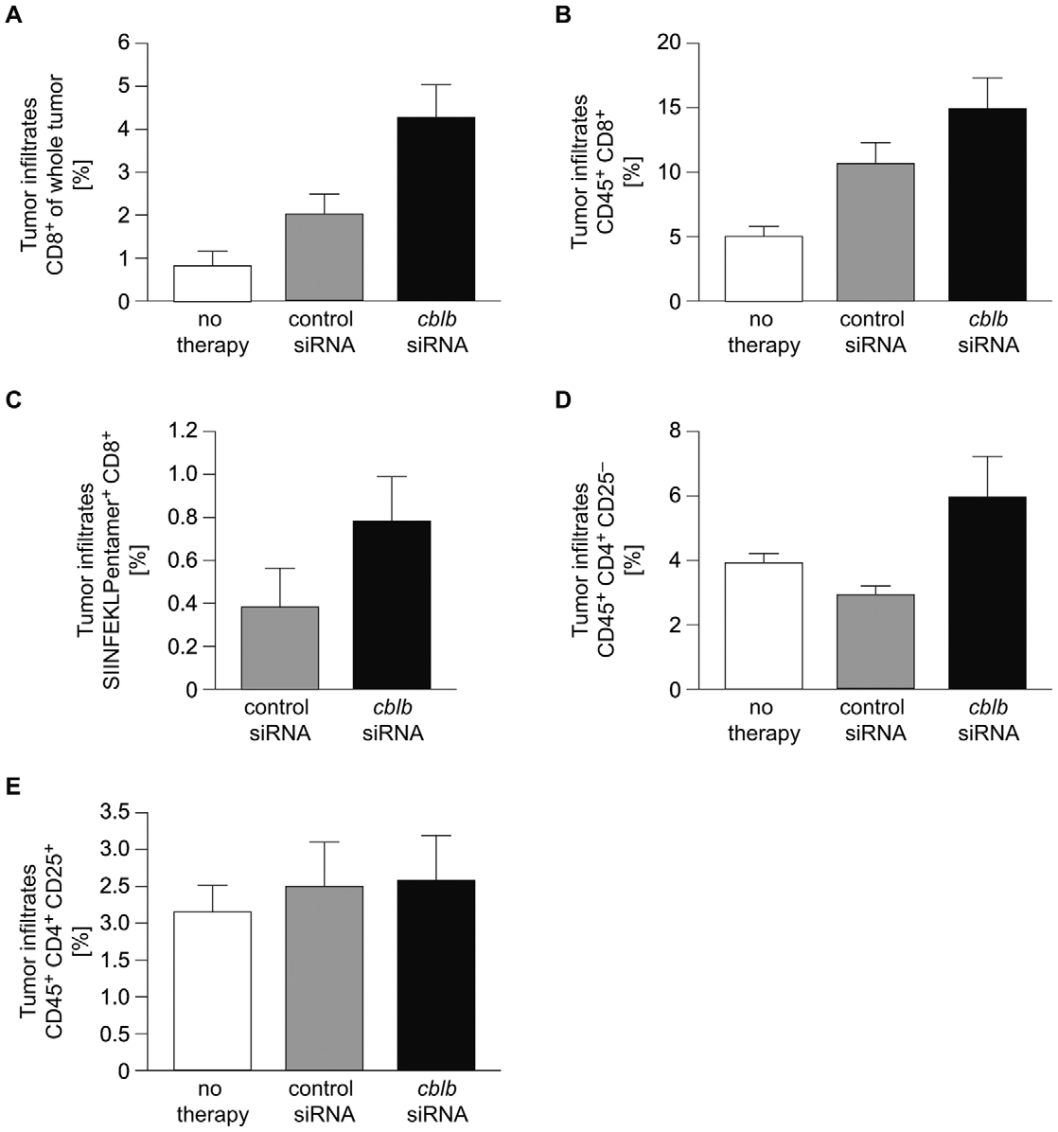
*cblb* silencing substantially enhances tumor infiltration of T cells. Flow cytometric analysis of tumor-infiltrating lymphocytes 7 days (A, B, D, E) or 4 days (C) after a single ACT in combination with DC vaccination into wild type mice was performed. (A) CD8^+^ T cell infiltrates gated on whole tumor, (B) CD8^+^ T cell infiltrates gated on total CD45^+^ leukocytes, (C) SIINFEKL-specific infiltrates gated on CD8^+^ cells (D) CD4^+^CD25^-^ T cell infiltrates and (E) CD4^+^CD25^+^ T cell infiltrates gated on total CD45^+^ leukocytes are shown. Means ± SEM of two independent experiments are shown (n = 7 - 8 mice per group). (A) p = 0.06 (DC+CD8^+^ *cblb* siRNA versus DC+CD8^+^ control siRNA) (B-E) n.s. (DC+CD8^+^ *cblb* siRNA versus DC+CD8^+^ control siRNA).

To assess whether silencing *cblb* in non-TCR-transgenic (polyclonal) CD8^+^ T cells would increase their ability to infiltrate and reject tumors, we employed ACT in an *in vivo* mouse B16ova melanoma model (Figure 2A). Considering the most efficient silencing oligonucleotide, we selected *cblb* siRNA #5 for the following experiments. As recipients, fully immune-competent C57BL/6 mice were used. Due to the reported insufficient therapeutic efficacy of *cblb* ^−/−^ CD8^+^ T cell ACT alone [21], we employed a combined treatment with a DC vaccine to induce *in vivo* selection of tumor antigen-specific CD8^+^ T cells. While at day 24 all mice in the untreated group had to be sacrificed due to large tumor size (Figure 2B), treatment with SIINFEKL-loaded DCs substantially delayed tumor outgrowth (Figure 2C). ACT of polyclonal CD8^+^ T cells treated with a non-silencing siRNA combined with DC vaccination resulted in no further improvement (Figure 2D). In contrast, combination of DC vaccination with ACT of *cblb*-silenced CD8^+^ T cells resulted in strong suppression of tumor growth, demonstrating that *cblb*-silencing in ACT can induce profound anti-tumor immune effects (Figure 2E). As an additional parameter for the efficacy of the *cblb* silencing in ACT, we determined overall survival after two treatment cycles. Although the combination therapy delayed tumor outgrowth and substantially enhanced overall survival (Figure 2F), all mice eventually succumbed to disease, in line with the transient nature of the silencing. Interestingly, a third treatment cycle (day 20/21) further prolonged the survival of tumor bearing mice (Table 1). To extend these findings to a more physiological setting, we vaccinated B16ova challenged mice with DCs loaded with the established melanoma antigen gp100 instead of SIINFEKL [26]. Combined with ACT of *cblb*-silenced CD8^+^ T cells, tumor outgrowth was again substantially suppressed (Table 2), and survival significantly prolonged (Figure 2G).

**Figure 4:**
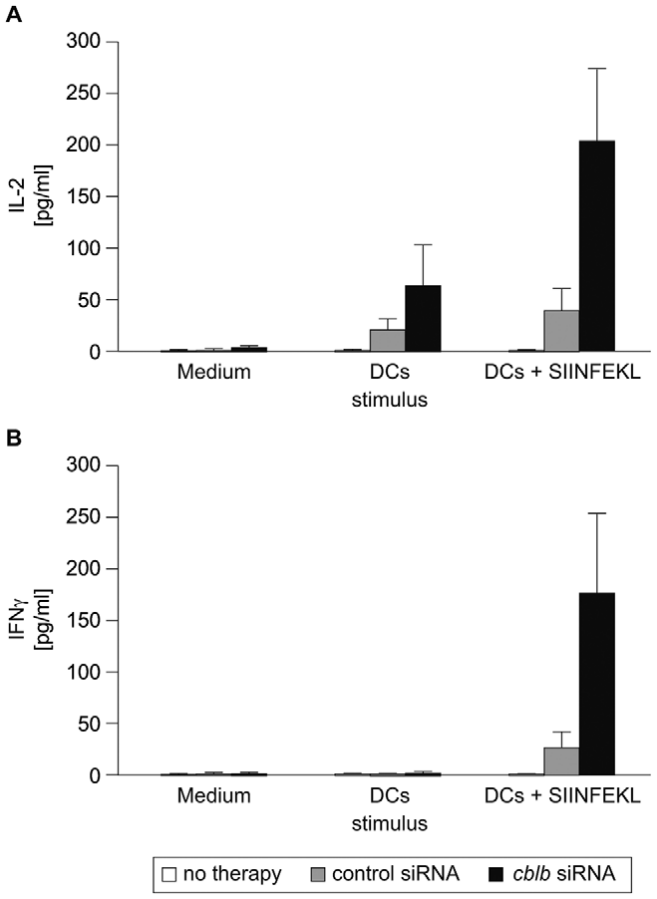
Enhanced cytokine recall responses of *cblb*-silenced CD8^+^ T cell tumor infiltrates. Tumor infiltrating CD8^+^ T cells either nucleofected with control siRNA or *cblb* siRNA were *ex vivo* stimulated with unloaded or SIINFEKL loaded DCs and (A) IL-2 as well as (B) IFNγ secretion was measured by BioPlex technology. Means ± SEM of four independent experiments are shown. (A) p = 0.03 (SIINFEKL loaded DC+CD8^+^ *cblb* siRNA versus SIINFEKL loaded DC+CD8^+^ control siRNA), (B) p = 0.05 (SIINFEKL loaded DC+CD8^+^ *cblb* siRNA versus SIINFEKL loaded DC+CD8^+^ control siRNA).

As a consequence of the enhanced immune response induced by the combination of DCs plus *cblb*-silenced ACT, we detected increased CD8^+^ T cell infiltration at the tumor sites (Figure 3A, B). Of note, the fraction of vaccine antigen-specific *cblb*-silenced CD8^+^ T cells in the tumor was twice as high compared to the control group (Figure 3C). Interestingly, although only CD8^+^ T cells were transferred, we also found a relative increase in CD4^+^ T cell infiltration (Figure 3D). In contrast, we could not detect any significant alteration in regulatory CD4^+^ CD25^+^ T cell infiltration (Figure 3E).

To evaluate the functional activity of intratumoral CD8^+^ T cells, we performed recall assays. One week after ACT, tumor infiltrating CD8^+^ T cells were isolated and *ex vivo* stimulated with antigen-pulsed DCs. CD8^+^ T cells isolated from mice treated with DCs combined with *cblb*-silenced ACT were hyperresponsive, as revealed by significantly enhanced cytokine secretion (Figure 4A, B). Taken together, siRNA-mediated silencing of *cblb* augments effector functions and infiltration rates of adoptively transferred CD8^+^ T cells, resulting in substantial suppression of tumor growth when transferred into tumor bearing mice vaccinated with a DC vaccine.

### ACT of *cblb* siRNA-treated CD8^+^ T cells in Combination with DC Vaccines does not Induce Symptoms of Autoimmunity in Recipient Mice

**Figure 5:**
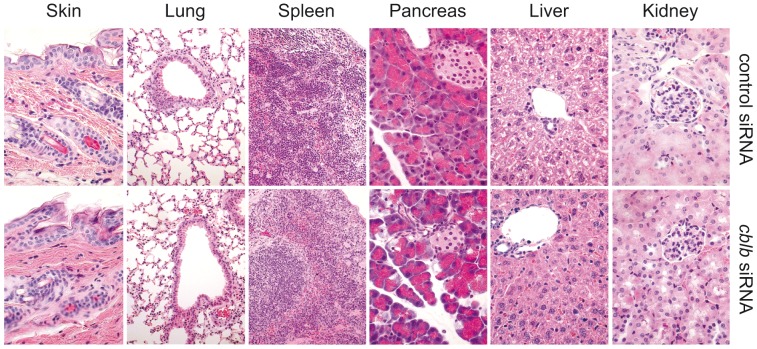
No overt morphological signs of autoimmunity in mice treated with *cblb*-silenced CD8^+^ T cells. B16ova tumor bearing mice were treated twice with SIINFEKL-pulsed DCs plus CD8*^+^* T cells either nucleofected with *cblb*- or control-siRNA. Animals were sacrificed when tumor length reached 2 cm. Histological sections of paraffin-embedded organs (skin, lung, spleen, pancreas, liver, and kidney) were stained with hematoxylin-eosin (HE) to analyze morphological signs of autoimmunity as well as immune cell infiltrates (n = 4) by an experienced pathologist in a blinded fashion.

Since *cblb* ^−/−^ mice have increased susceptibility to autoimmunity [12], we next investigated whether mice treated twice with *cblb*-silenced CD8^+^ T cells in combination with DC vaccines would suffer from autoimmune side effects. This possibility arises from the observation that anti-tumor T cell responses can be directed to non-vaccine antigens in a process termed "antigen spreading" [27]. Because we were using polyclonal rather than antigen-specific CD8^+^ T cells for ACT, T cell responses towards self-antigens could be principally conceivable. However, despite the clear anti-tumor immune responses, no detectable clinical or morphological signs of autoimmunity were observed (Figure 5). This positive safety assessment of *cblb* knockdown CD8^+^ T cell ACT suggests that autoimmune side effects of the therapy might not hamper a potential clinical translation of the concept.

### siRNA Mediated Silencing of *CBLB* is Effective and Leads to Hyper-responsiveness in Human T cells

**Figure 6:**
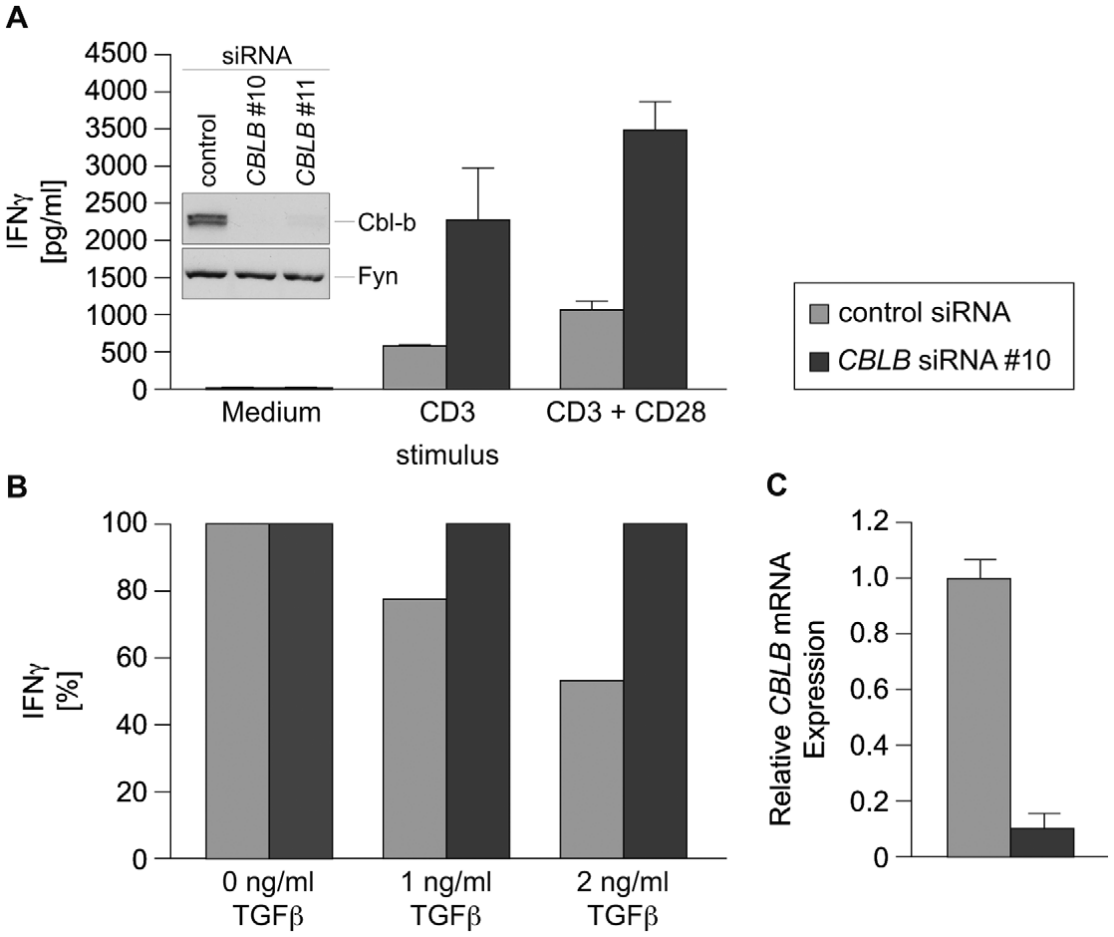
siRNA-mediated silencing of human *CBLB* is effective and leads to hyper-responsiveness of CD8^+^ T cells. Human CD8^+^ T cells were nucleofected with *CBLB* siRNA #10 or control siRNA and stimulated with anti-CD3 and anti-CD28 as indicated. Similar results were obtained with non-overlapping siRNA oligonucleotide #11 (not shown). (A) Supernatants were taken two days post-transfection and the amount of IFNγ secreted was measured by BioPlex technology. Means ± SEM of two independent experiments are shown. p = 0.01 (CD8^+^ *CBLB* siRNA versus CD8^+^ control siRNA, double stimulated). (B) Human CD8^+^ T cells were nucleofected with *CBLB* siRNA or control siRNA and stimulated with anti-CD3 and anti-CD28 in the presence or absence of TGFβ. Supernatants were taken two days post-transfection and the amount of IFNγ secreted was measured by BioPlex technology. IFNγ levels of each group without TGFβ were set 100%. Representative results of two independent experiments are shown. Silencing efficacy of *cblb* siRNA was analyzed by (A) immunoblotting (One experiment representative of three experiments is shown) or (C) qRT-PCR. Means ± SEM of two independent experiments are shown.

To transfer this approach into the human setting, we established a similar procedure for *ex vivo* silencing of *CBLB* in human CD8^+^ T cells. As expected, *CBLB* knockdown resulted in efficient downregulation of Cbl-b protein levels. In agreement with the phenotype observed in murine T cells, *CBLB* silencing in human CD8^+^ T cells also significantly enhanced IFNγ production, even in the absence of CD28 costimulation (Figure 6A). Additionally, *CBLB*-silenced human CD8^+^ T cells were markedly less susceptible to the inhibitory effects of TGFβ (Figure 6B). Of note, *CBLB* mRNA was almost undetectable even on days 5 and 7 after transfection (Figure 6C and not shown). This suggests that in a therapeutic setting, anti-tumor CD8^+^ T cells should be hyperreactive for at least several days.

## Discussion

To date, there is limited evidence for a broad therapeutic efficacy of adoptive T cell therapy for cancer in the clinical setting. Lowering activation thresholds and/or circumventing immunosuppressive tumor milieu signals that interfere with T cell effector functions are therefore valuable approaches to increase the effectiveness of adoptive T cell therapies. Along this line, the co-receptor cytotoxic T lymphocyte–associated antigen 4 (CTLA4) is pivotal in regulating the threshold of CD28 costimulatory signals during T cell activation [28]. Consistently, CTLA4 blockade with the CTLA4–specific antibody ipilimumab™ leads to increased T cell-mediated effector responses and induces cancer regression in clinical trials of patients with metastatic melanoma [29]. However, this approach also causes immune-related toxicity [30], [31]. Of note, CTLA4 and CD28 control T cell activation via a Cbl-b dependent mechanism [32]. Targeting Cbl-b in peripheral T cells may provide an alternative strategy to enhance the efficacy of adoptive T cell immunotherapies. Additionally, the tumor microenvironment, and particularly TGFβ mediated suppressive signals, are among the most important cancer-induced immune evasion signals inhibiting effector T cell populations. Cbl-b is an established regulator of TGFβ sensitivity; TGFβ-induced inhibitory effects on T cells can be attenuated by loss of *cblb* expression [19]. Consistently, targeting Cbl-b should confer, at least partially, resistance of transferred effector T cell populations towards these suppressive milieu effects.

The present study strongly supports this hypothesis and demonstrates for the first time that *cblb* gene silencing in CD8^+^ T cells prior to adoptive T cell therapy efficiently delays tumor outgrowth. The *cblb*-silenced ACT mouse group showed increased CD8^+^ intratumoral infiltration and significantly improved survival. Due to the reported insufficient therapeutic efficacy of *cblb* ^−/−^ CD8^+^ T cell therapy alone in the B16 melanoma model using polyclonal T cells ([21] and data not shown), we combined ACT with a DC vaccine to induce an efficient *in vivo* selection of tumor antigen-specific T cells. In this mouse tumor model, siRNA-mediated *cblb* silencing and repetitive ACT act as a potent adjuvant for DC vaccination and provide a significant therapeutic benefit. It is noteworthy that we did not use TCR-transgenic tumor-antigen specific but polyclonal T cells for ACT. Moreover, in our system ACT was not combined with lymphopenia as approached in a magnitude of pre-clinical models. Overall, our findings suggest that destroying tumors via synthetic *cblb* siRNA-silenced CD8^+^ T cells might represent a treatment option for human cancer therapy.

Taken together, Cbl-b governs the threshold for T cell activation by regulating both TCR/CD28 signaling and TGFβ sensitivity of T cells in adoptive immunotherapies. We here provide proof of concept that synthetic siRNA-mediated inactivation of *cblb* in polyclonal CD8^+^ T cells may improve the efficacy and broaden the applicability of adoptive T cell therapy. The validity of this hypothesis and its clinical implication remain to be seen.

## Materials and Methods

### Mice

Mice were maintained under specific pathogen-free conditions. All experiments were performed in accordance with protocols approved by the Institutional Animal Care and Use Committee.

### Medium, Reagents, and Antibodies

RPMI medium, fetal calf serum, trypsine, and G418 were obtained from Biochrom, L-glutamine, penicillin/streptomycin from Gibco, and 2-mercaptoethanol from Sigma. The SIINFEKL peptide was from Polypeptide Laboratories and the gp100 peptide [33] was a kind gift of Patrizia Stoitzner, Austria. Lipopolysaccharide was from Sigma-Aldrich. Anti-CD3 (clones 2C11 and OKT3) was made in-house and murine and human anti-CD28 was obtained from BD Pharmingen. TGFβ was from eBiosciences. For FACS analysis, anti-CD4, anti-CD8α, and anti-CD25 from BD Pharmingen and anti-CD45 from eBiosciences were used. SIINFEKL-Pentamer was from Proimmune. Anti-Cbl-b (sc-8006) and anti-Fyn (sc-16) were from Santa Cruz. *cblb* siRNAs were from Dharmacon RNA Technologies (Mouse #5∶5'-AAAUUCUCGAAGUAUGCUCUU-3′, Mouse #6: UAACUUCCAGGCUUGGUGCUU-3′, Human #10 5'-UUUGCUAACGGACCAGUACUU-3′ and Human #11 5'-UAAUACCCAAAAUUCGACCUU-3′ and On-Target plus siRNA control #1).

### Cell Lines and Tumor Model

1×10^5^ B16ova cells [34] (provided by Drs. R Kemp and D Dutton, Trudeau Institute, NY, USA) were injected subcutaneously into the flank of 6–8 week old female recipients. Tumor growth was monitored by caliper-measured tumor length and width. Tumor volume was calculated according to the following equation: (length × width^2^) × (π/6). For survival analysis, mice with tumors above the length limit of 20 mm were sacrificed.

### Electroporation of Primary CD8^+^ T cells

Delivery of chemically synthesized short interfering RNA (siRNA) into CD8^+^ T cells was accomplished using the Amaxa Nucleofector system and T cell Nucleofector Kits (Lonza) according to the manufacturer’s recommendations. 1×10^7^ cells were transfected with 1.5 µM siRNA and programs X-01 for mouse and V-24 for human CD8^+^ T cells were used, respectively. After transfection, cells were rested for 1 h in pre-warmed Nucleofector medium at 37°C and 5% CO_2_ prior to ACT, or, alternatively, cultured for a minimum of 24 h before further *in vitro* analyses.

### CD8^+^ T cell and DC Preparation

Human CD8^+^ T cells were derived from peripheral blood lymphocytes and murine CD8^+^ T cells were purified from the spleen and lymph nodes by negative selection using magnetic beads (Miltenyi Biotech). Dendritic cells (DCs) were generated from total bone marrow cells from wild type animals using RPMI supplemented with 10% fetal calf serum, 2 mM L-glutamine, 100 U/ml penicillin, 0.1 mg/ml streptomycin, 0.05 mM β-mercaptoethanol, and 200 U/ml granulocyte-monocyte colony-stimulating factor (GM-CSF), obtained from supernatant of X38-Ag8 plasmacytoma cells stably transfected with the murine *GMCSF* gene [35] (gift of A Lanzavecchia, Bellinzona, Switzerland). On day 6, DCs were loaded with 10 µM SIINFEKL or gp100 peptide and stimulated with 100 ng/ml lipopolysaccharide for 4 h.

### DC Vaccination and Adoptive Cell Transfer

2×10^5^ SIINFEKL-loaded or gp100-loaded DCs were subcutaneously injected into the contralateral left flank of tumor-bearing mice on days 6 and 13 after tumor challenge. Adoptive cell transfer (ACT) was performed on days 7 and 14 by injecting 5×10^6^ polyclonal CD8^+^ T cells via intravenous tail vein injection.

### FACS Analysis

Single cell suspensions of tumor tissue were stained with anti-CD4, anti-CD8, anti-CD25, anti-CD45 and SIINFEKL-Pentamer and analyzed on a FACSCalibur (BD Biosciences).

### Recall Assay

CD8^+^ T cells were purified from single tumor cell suspensions 7 days after a single ACT at day 14 by two rounds of positive selection using magnetic beads (Miltenyi Biotech). CD8^+^ T cells were restimulated with SIINFEKL loaded DCs at a ratio of 2∶1. Supernatants were collected after 24 h and analyzed by BioPlex technology (Biorad) for the presence of IL-2 and IFNγ.

### Analysis of TGFβ Sensitivity

Isolated human or mouse CD8^+^ T cells were nucleofected with either control or *cblb* siRNA. After resting over night, 5×10^5^ cells were stimulated with 5 µg/ml plate-bound anti-CD3 mAb together with 1 µg/ml soluble anti-CD28. TGFβ was added as indicated. Supernatants were collected on day 1 or day 2 and analyzed by BioPlex technology for the presence of IFNγ.

### Quantitative RT-PCR

RNA was isolated with the MagAttract direct mRNA M48 kit (Qiagen). cDNA was synthesized with the Qiagen Omniscript RT kit according to manufacturer’s protocol. Expression of *CBLB* was quantified via RT-PCR on an ABI PRIM 7000 Sequence Detection System (Applied Biosystems) using a TaqMan gene expression assay (Hs00180288_m1). Expression was normalized for GAPDH.

### Statistical Analysis

For statistical analysis, Student's *t*-test was used. p-values ≤0.05 were considered significant. Figures show means ± SEM. Overall survival is expressed by the Kaplan–Meier method and differences between groups were determined with the log-rank test. Statistical analysis was performed using SPSS.
